# Roles of Aquaporins in Plant-Pathogen Interaction

**DOI:** 10.3390/plants9091134

**Published:** 2020-09-01

**Authors:** Guangjin Li, Tong Chen, Zhanquan Zhang, Boqiang Li, Shiping Tian

**Affiliations:** 1 Key Laboratory of Plant Resources, Institute of Botany, Innovation Academy for Seed Design, Chinese Academy of Sciences, Beijing 100093, China; liguangjin@ibcas.ac.cn (G.L.); chentong@ibcas.ac.cn (T.C.); zhangzhanquan82@ibcas.ac.cn (Z.Z.); bqli@ibcas.ac.cn (B.L.); 2 University of Chinese Academy of Sciences, Beijing 100049, China

**Keywords:** aquaporins, plant immunity, pathogenicity, plant-pathogen interaction

## Abstract

Aquaporins (AQPs) are a class of small, membrane channel proteins present in a wide range of organisms. In addition to water, AQPs can facilitate the efficient and selective flux of various small solutes involved in numerous essential processes across membranes. A growing body of evidence now shows that AQPs are important regulators of plant-pathogen interaction, which ultimately lead to either plant immunity or pathogen pathogenicity. In plants, AQPs can mediate H_2_O_2_ transport across plasma membranes (PMs) and contribute to the activation of plant defenses by inducing pathogen-associated molecular pattern (PAMP)-triggered immunity and systemic acquired resistance (SAR), followed by downstream defense reactions. This involves the activation of conserved mitogen-activated protein kinase (MAPK) signaling cascades, the production of callose, the activation of *NPR1* and *PR* genes, as well as the opening and closing of stomata. On the other hand, pathogens utilize aquaporins to mediate reactive oxygen species (ROS) signaling and regulate their normal growth, development, secondary or specialized metabolite production and pathogenicity. This review focuses on the roles of AQPs in plant immunity, pathogenicity, and communications during plant-pathogen interaction.

## 1. Introduction

Aquaporins (AQPs) are membrane channel proteins that are primarily associated with water transport across cell membranes [1]. Water transportation is extremely important for all living cells to maintain cellular functions and normal vital activities under various conditions. Less than 30 years after the discovery in human red blood cell membranes, AQPs are now known to exist in nearly all living organisms, suggesting their essential role in physiological functions [1,2,3]. In addition to water, some AQPs can also transport small solutes (including urea, ammonium, arsenite, lactic acid, boric acid, and glycerol), micronutrients (including silicon and boron), other small molecules (reactive oxygen species, ROS), and even gas molecules (including CO_2_, O_2_ and NO), some of which may function as crucial signaling molecules during various cellular responses under stress conditions [4,5,6,7,8,9,10]. In contrast, non-transporting functions of some AQPs include cell-cell adhesion, membrane polarization, and regulation of interacting proteins, such as ion channels [1]. Compared to the functions of AQPs in symbiotic plant-microbe interaction, it has become increasingly clear that AQPs also play an important role in host-pathogen interaction. The present review focuses on the roles of AQPs in plant immunity, pathogen pathogenicity, and communications during pathogenic plant-microbe interaction.

The numbers of *AQP* genes vary significantly among different species [11]. Recent genomic sequencing projects have shown that AQPs are more abundant in eukaryotes as compared to prokaryotes [12]. To date, at least 35, 36, 33, 70, 47 aquaporin genes of higher plants have been identified in *Arabidopsis*, maize, rice, cotton, and tomato, respectively [13,14,15,16,17]. The first reported AQP in the plant was AtTIP1;1, a tonoplast intrinsic protein from *Arabidopsis*. Its functions have been further analyzed through expression studies in *Xenopus* oocytes and cell-swelling experiments in hypoosmotic media [18]. Since their discovery in the 1990′s, numerous AQPs have been identified and investigated in plants. Based on their sequence similarity and specific subcellular localization, AQPs in the plant are divided into five major subgroups, including the plasma membrane (PM) intrinsic proteins (PIPs), X intrinsic proteins (XIPs), and nodulin 26 like intrinsic proteins (NIPs) in the PM, tonoplast intrinsic proteins (TIPs) in tonoplast, and small basic intrinsic proteins (SIPs) in the endoplasmic reticulum [19,20]. Each subfamily can be further divided into different subgroups according to their specific locations and functions. For example, PIPs have been classified into two subgroups, namely, the PIP1 subfamily composed of PIP1;1 to PIP1;5 and the PIP2 subfamily composed of PIP2;1 to PIP2;8 [21]. PIPs are mainly in charge of substrate transport between the exterior and interior of cells, whereas the others function in transport between organelles [22,23]. Gene knockout studies have revealed that AQPs participate in regulating the many physiological processes in plants, including water uptake, gas exchange, nutritional elements and heavy metal acquisition, seed formation and germination, calcium, and ROS-mediated signaling and biotic and abiotic stresses responses [19,21]. Some microbes, such as bacteria, show less aquaporin diversity, typically possessing only one or two *AQP* genes, and the absence of such genes has not revealed any definite phenotype [1]. Moreover, AQP-deletion mutants have also been studied in *Botrytis cinerea* and *Fusarium graminearum*, respectively, suggesting that AQPs also have important roles in growth, development, secondary metabolism, and pathogenicity of fungal pathogens [5,24].

AQPs are tightly controlled through multiple mechanisms, mostly including transcriptional control of their expression and post-translational modifications to control their abundance and transport activity [25,26]. Many reports suggest that AQPs are upregulated or downregulated in plants in response to environmental cues [27,28,29]. Nevertheless, the post-translational regulation (such as phosphorylation, methylation, deamidation, and acetylation) that regulates PM delivery and the activity of PIPs is still unexplored [19,30]. These regulation mechanisms can influence the conformation of AQP monomers, their stability in PMs, and their trafficking or subcellular localization [26,31]. Phosphorylation is a common mode of post-translational modification that acts as a molecular ‘switch’ to regulate protein activity in response to various stresses. It has been proven that phosphorylation of AtPIP2;1 at multiple sites in the C-terminal occurs under salt stress conditions, leading to the switch of AtPIP2;1 from PMs to intracellular regions, reducing the hydraulic conductivity of *Arabidopsis* [32].

## 2. The Function of Aquaporins in Plant-Pathogen Interaction

Plants are constantly under attack by pathogens, including viruses, bacteria, and fungi, which leads to various diseases in economically important plants and significant economic losses [33,34]. Plant pathogens have developed efficient strategies to attack the hosts, whereas plants also employ functional innate immune systems for defenses against pathogens [35]. The PM is one of the first compartments where plant-pathogen interaction occurs, which mainly depends on the functions of membrane proteins and other biomacromolecules. Increasing evidence suggests that AQPs play key roles in plant-pathogen interaction involved in plant immunity and pathogen pathogenicity.

### 2.1. AQPs Regulation of Plant Immunity

To protect themselves against attack by pathogens, plants perceive conserved pathogen-associated molecular patterns (PAMPs) from various pathogens, such as bacterial flagellin, harpin or fungal chitin, which further trigger PAMP-triggered immunity (PTI) and activate various defense reactions, including the production of ROS, the activation of ion fluxes and conserved mitogen-activated protein kinase (MAPK) signaling cascades, the secretion of antimicrobial secondary metabolites, stomatal closure, and cell wall strengthening [36,37]. Pathogens can suppress PTI by delivering effector proteins into plant cells, resulting in successful infection. In turn, plants have developed another stronger and faster immunity response called effector-triggered immunity (ETI), which involves localized cell death, also termed hypersensitive response (HR) and systemic acquired resistance (SAR) in the whole plant to limit pathogen spreading [38,39].

**Figure 1:**
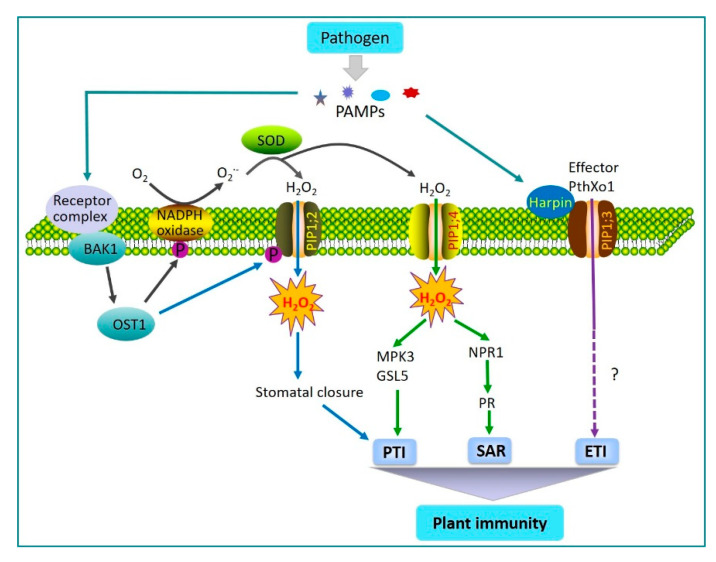
A working model for the involvement of aquaporins in mediating plant immunity. The perception of pathogen-associated molecular patterns (PAMPs) leads to the activation of the immune receptor complex. Brassinosteroid insensitive 1-associated receptor kinase 1 (BAK1) and open stomata 1 (OST1) may further activate membrane-localized NADPH oxidase and PIP1;2. Phosphorylation at Ser121 in PIP1;2 by BAK1 and/or OST1 activates the H_2_O_2_ transport activity of PIP1;2, further inducing stomatal closure and leading to PAMP-triggered immunity (PTI). Alternatively, PIP1;4 is also involved in the transport of apoplastic H_2_O_2_ into the cytoplasm, further orchestrated by SAR or PTI, to counteract pathogen invasion. In addition, PIP1;3 directly interacts with harpin Hpa1 at the PM to mediate the translocation of the effector PthXo1 into host cells.

The oxidative burst is an early defense reaction of plants to pathogen attack, leading to the generation of ROS around the infection site [40,41]. The rapid and transient generation of ROS, particularly H_2_O_2_, reflects a successful recognition of pathogen invasion, which further triggers a variety of immune responses, such as PTI and SAR, to regulate plant disease resistance [42]. The PM-localized NADPH oxidase is a main factor responsible for the PAMP-induced ROS burst in plants, which catalyzes the production of superoxide by transferring electrons from cytosolic NADPH to apoplastic oxygen, and finally, the production of H_2_O_2_ by superoxide dismutases [43]. Apoplastic H_2_O_2_ is then rapidly translocated into the cytoplasm across the PM. Recently, it has been demonstrated that AtPIP1;4, one of the PIP family members in *Arabidopsis*, was involved in the transport of H_2_O_2_ across the PM [44]. Meanwhile, several downstream defense reactions were observed following this process, including the activation of conserved MAPK signaling cascades by inducing the expression of *MPK3*, the production of callose by inducing the expression of *GSL5*, and the activation of *NPR1* and *PR* genes [44] (Figure 1). In AtPIP1;4 knockout mutants, H_2_O_2_ was prevented from being transported into the cells, and high concentrations of H_2_O_2_ accumulated within apoplastic regions, leading to hypersensitivity to the pathogen. These findings indicated AtPIP1;4 in *Arabidopsis* links the translocation of apoplastic H_2_O_2_ to the activation of the PTI and SAR pathways in response to PAMPs. Furthermore, several other AQPs in plants, including the PIPs and TIPs, were also shown to mediate H_2_O_2_ transport across the membrane between cells [45,46]. Importantly, the structural configuration may greatly facilitate the elucidation of the functions of AQPs. A recent, excellent review has provided some details on the structural features of AQPs for regulating H_2_O_2_ transport, which may add more values to the understandings of sophisticated mechanisms involving AQPs in host-pathogen interaction [22]. In turn, H_2_O_2_ also regulates the expression, activity, and localization of specific AQPs and affects the capacity of AQPs to transport H_2_O_2_, thus contributing to H_2_O_2_-induced immune responses in plants [47,48,49].

As a part of plant innate immunity, plants have the capacity to close their stomata after the perception of PAMP to restrict pathogen invasion [50,51]. Stomatal opening and closing are precisely controlled by various endogenous and environmental stimuli [50]. It has been known that the stress hormone ABA participates in the regulation of stomatal closure [52]. AtPIP1;2 was found to facilitate the transport of water and H_2_O_2_ across the PM of guard cells to trigger ABA- and pathogen-induced stomatal closure in *Arabidopsis* [50]. Phosphorylation of AtPIP1;2 at Ser121 by Brassinosteroid Insensitive 1-associated Receptor Kinase 1 (BAK1) and/or Open Stomata 1 (OST1) may activate H_2_O_2_ transport activity of AtPIP1;2 in response to ABA signaling and PAMP recognition (Figure 1). These results clearly showed that AQPs were involved in the opening and closing of stomata leading to PAMP-triggered immunity.

Harpin is a PAMP elicitor secreted by the type III secretion system in pathogenic bacteria [53]. It has the ability to create pores through cell membranes by directly binding to membrane components, and thus, promoting the translocation of effector proteins into plant cells [54]. Recent results indicated that the translocator function of bacterial harpin protein Hpa1 depended on their interaction with aquaporin PIP1;3 in rice, which participated in the regulation of PthXo1 effector translocation [55] (Figure 1). Knockout or overexpression of *PIP1;3* did not affect Hpa1-induced immune responses, but substantially affected the susceptibility of rice to bacterial blight pathogen [55,56]. In *A. thaliana*, Hpa1 also interacted with the H_2_O_2_ transport channel AtPIP1;4, which facilitated apoplastic H_2_O_2_ entry into the cytoplasm and may lead to the initiation of an immune response [22,57]. There is no evidence to indicate whether *PIP1;2* participates in effector translocation that needs further investigation.

Evidence for the participation of AQPs in plant immunity has been drawn from the transcript profiling of plants infected by pathogens [29]. In addition to increased expression of defense-related genes, a decline in the expression of a number of aquaporin-encoding genes was observed in cotton [29]. In citrus plants and soybean leaves, several *AQPs* were also found to be differentially expressed exclusively following the pathogen infection—indicating that these genes may be involved in disease development [58,59]. Based on the above observations, further studies are required to determine whether these AQPs have a specific role in plant resistance to the pathogen. In addition, it is noteworthy that a number of other solutes or gases, such as nitric oxide and silicon, have been reported to be transported across the membrane via AQP channels. In contrast, these small molecules are involved in a variety of metabolic processes or function associated with plant immunity [4,10].

### 2.2. AQPs Regulation of Fungal Pathogen Pathogenicity

Plant fungal pathogens efficiently colonize the hosts to obtain the necessary nutrients for their growth and survival [33]. During the initial phases of infection, fungal pathogens regulate their growth and development tightly and develop specialized infection structures (such as appressoria, infection cushions, and hyphae) for penetrating hosts and absorbing nutrients [60]. To further colonize hosts and cause disease, fungal pathogens usually produce and secrete a number amount of extracellular virulence factors, including effectors, ROS, and toxic secondary metabolites [33,61]. Effectors can directly suppress host immunity, while toxic secondary metabolites and ROS may contribute to killing host cells.

**Figure 2:**
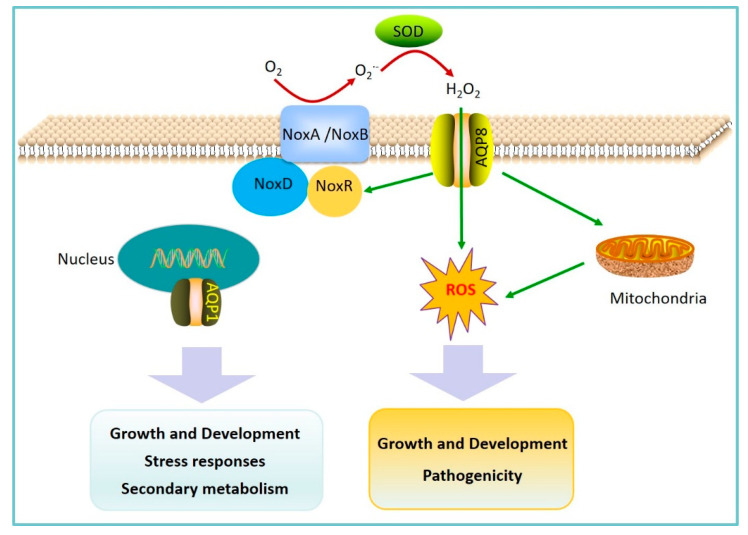
A working model for the involvement of aquaporins in modulating pathogen pathogenicity. As important second messengers, ROS are generally produced in mitochondria or at PM, in which NADPH oxidase (Nox) complexes have crucial functions. Aquaporin8 (AQP8) is involved in ROS production by influencing *noxR* expression, mitochondrial distribution, and H_2_O_2_ transport across membranes. Consequently, ROS-mediated signaling is activated and functions in regulating the growth, development, and pathogenicity of pathogens. In addition, AQP1 is localized to the nuclear membrane and plays an important role in hyphal growth, sexual and asexual development, stress responses, and secondary metabolism.

Fungal pathogens have the capability to produce various small molecules called secondary metabolites, which have been shown to function as virulence factors in the interactions with plants [62]. Fungal pathogens secrete toxic secondary metabolite inducing programmed cell death of host cells [61]. Deoxynivalenol (DON) is an important trichothecene mycotoxin and virulence factor produced by several *Fusarium* spp. [63]. DON can interfere with normal cellular functions through inhibition of protein translation by binding to the ribosomes. In *F. graminearum*, the AQP protein FgAQP1 localized at the nuclear membrane was crucial for hyphal growth, sexual and asexual development, stress responses, and secondary metabolism [24]. The deletion of *FgAQP1* significantly affected DON production and the expression of related genes, indicating that FgAQP1 may play key roles in *F. graminearum*-host interaction (Figure 2).

ROS are known to be involved in plant defense, but also for pathogen attack. Increasing evidence indicated that fungal pathogens could also generate ROS as signaling components, which are important for hyphal growth, development, infection structure formation, and fungal pathogenicity [64,65]. Similar to plants, the primary enzymatic ROS generating systems in fungal pathogens is the NADPH oxidase complex, and mitochondria are major sources of intracellular ROS [66,67,68]. *B. cinerea* is an economically important necrotrophic fungal pathogen that can cause devastating diseases, especially on fresh fruits and vegetables. An et al. identified eight AQP genes in *B. cinerea*, among which the only AQP8 participates in ROS production, distribution, and transport across the PM [5] (Figure 2). It has been proven that specific AQPs can regulate H_2_O_2_ membrane permeability and signaling and facilitate the transport of H_2_O_2_ across the PM in living organisms [69]. These studies indicated that AQP8 could mediate H_2_O_2_ uptake through its capacity to act as a membrane channel for H_2_O_2_, and therefore, affect the ROS signal pathway. In *B. cinerea*, the deletion of *AQP8* obviously suppressed the expression of the *noxR* gene, indicating that AQP8 may affect the function of the NADPH oxidase complex and the production of ROS. In addition, the disruption of both *AQP8* and *noxR* changed the distribution of mitochondria in *B. cinerea* hypha. Moreover, *AQP8* disruption significantly impaired mycelial growth, conidiation, infection structures formation, and virulence. The results imply that AQP8 was indispensable for normal growth, development, and pathogenicity through their mediation of ROS signaling transduction in *B. cinerea*.

## 3. Prospects for Future Research

AQPs are membrane channel proteins that primarily transport water and small solutes across membranes in nearly all living organisms [1]. Recent studies have shown that AQPs play pivotal roles both in plant immunity and pathogen pathogenicity during plant-pathogen interaction. Although diverse classes of AQPs in plants are differentially regulated upon pathogen attack, their roles, especially the intracellular AQPs (such as TIPs, NIPs, and SIPs), in plant-pathogen interaction, are largely unknown. Apart from expression analysis, abundance, activity, gating, trafficking, and subcellular relocalization of AQPs should be further evaluated by integrating physiological, biochemical, and molecular genetic methods. Notably, plants may employ tissue- or cell type-specific *AQP* genes in different biological contexts, thus responding to diversified environmental conditions [70]. Further studies are still required to go into details to decipher the assembly of protein complexes and underlying mechanisms.

AQPs are tightly regulated at multiple levels in their expression, abundance, and transport activity, but the molecular mechanisms involving transcriptional, post-translational regulatory mechanisms, and molecular interactions need to be further deciphered. It may appeal to great interests to determine how AQPs are involved in endocytic activities and how effectors are simultaneously internalized with PM protein via membrane trafficking. Answers to these questions may provoke new ideas for efficiently protecting crops and controlling pathogens. Moreover, further characterization of upstream signaling events and their cross-talks also represents significant challenges for future research. Although accumulating evidence has shown that AQPs are indispensable for growth, development, and pathogenicity of pathogens, a more comprehensive understanding of the interacting partners and regulations on cellular redox homeostasis of AQPs still requires further investigation.
